# The Debateable Land in Psychology

**Published:** 1862-07

**Authors:** 


### Art. VIII.—THE DEBATEABLE LAND IN PSYCHOLOGY.

"They dwell in the bounds or meeting of the two kingdoms, but obey the laws of neither."—Fuller: *Worthies of England*, p. 216.

We all recollect the clumsy expedient of shutting in a few million miles of China by a wall, of the geographical puzzle involved in the Oregon treaty, and of the linear limitation of human goods and chattels by a degree in latitude on the suggestion of the Negrophiles. In early and simple forms of society the territories and rights of different nations were marked out and separated by a tract or strip of country which was claimed by the inhabitants of each of its borders, but actually belonged to neither; and was left bare and barren, and generally in its native savagery. We suspect that similar margins were sometimes left around smaller divisions of the earth's surface, as we encounter bits of isolated field, or furze, or peatland which still retain the sad cognomen of No-man's-land. Like the Corse-rig, they are sacred to mystery and to melancholy tradition to this day. These borders were invariably the appropriate region of myth and romantic superstition; and practically, they were the scene of incessant foray and fight, riot, robbery, and dispute. Those who dwelt near them slept in their spurs, herded their sheep with a spear in place of a crook. They were appropriately named the debateable lands.

There is, in the opinion of many, a similar tract connecting, or separating, the confines of sanity and insanity. Our maps, and the treaties, as they may be called, between philosophers and psychologists upon the one side, and the unenlightened classes upon the other, originally defined these limits sharply and distinctly. Mania and melancholia had a narrow, but precise and palpable boundary, and all beyond was intelligence and health—warped by crime, darkened by ignorance, or enfeebled by passion or age, it might be, but still responsible intelligence. There was no room left for doubt or dispute as to what appertained to the regions of folly, extravagance, and disease, and what legitimately fell under the dominion of reason and healthy judgment. There was, in fact, once a linear boundary between these as traceable, but it is suspected as imaginary, as what may be seen in a map of Africa surrounding the supposed realms of the King of Dahomey. But now vast spaces running parallel to, and on each side of this frontier are of doubtful ownership, pass alternately from the rule of one nominal sovereign to that of another, and then lapse back into a disputed allegiance. Eccentricity, feeblemindedness, and depravity are now sometimes classed as diseases, sometimes as delinquencies, and may consign a man by what appear very arbitrary laws, and which vary according to the time and the judge and the court, to a jail or an asylum. The uncertainty, or rather the many-sided nature of the subject, has suggested the suspicion that there is no such broad division between mental health and disease, that these states pass into each other by a gradation so slightly marked or so insensible, and modified by so many external and collateral circumstances, that in speaking of or dealing with their various forms, we have before us not separate entities, but different degrees or states of the same entity. It may be held, for example, that idiocy is not a privation but a rudimentary condition, the unevolved mental germ; that imbecility is a higher stage in the process of development; and that senile dementia is merely the old age of the mind, as enfeebled nutrition is that of the body. The controversy as to how far a fatuous person can be regarded as a lunatic is an illustration of the same view. The debate has arisen chiefly from the confusion, or rather the neglect, of nosology in medical nomenclature. Were fatuity or dementia, for they must be held as synonymous, defined enfeeblement of perception, or judgment, or memory, or of all of them in different degrees, a greater, if not an absolute unanimity of opinion might be expected. But when the dotage of old age, the impairment following apoplexy or epilepsy, the exhaustion accompanying rapid growth, the dulness characterising catamenial irregularities, and many states of imbecility are confusedly heaped together, it need not be matter for surprise that to many, even discriminating minds, the attempts to class all such affections under the same category appeared a gross and ignorant assumption. Yet in all the pathological condition was, perhaps, altered and defective nutrition in the nerve structure; in all the psychical nature of the alteration was weakening without perversion of the faculties; and the error consisted either in omitting all consideration of the theory of gradation or of the amount of enfeeblement in the different classes of cases: or in contemplating them through the character of their origin.

And to follow out the same train of thought, the diseases of the passions and appetites may be viewed as the merging of natural instincts and tendencies into excitement, extreme in nature and beyond the control of the will, either in consequence of the deficient exercise of that power itself, or of the reason which guides it.

Regarded as a distinct, definable condition, there may be no such thing as insanity at all. The inquiry is complicated by two considerations. In the first place, the fact is obtruded upon us by every contact with our fellow men of the vast and startling comparative differences in intellectual strength and in the acuteness of moral perceptions compatible with perfect mental health, and imposing the necessary rule of estimating soundness, or serenity, or responsibility in reference to the capacity of each mind, and not in reference to an abstract standard, nor to the minds of other individuals. And in the second place, there must be included in the estimate the different effects produced by the same circumstances upon different individuals, or upon the same individual at different times. It is even expedient to measure these manifestations in relation to position, education, age, and bodily health. What would be gross in the polished and pure, would be *gauche* in the vulgar clodhopper; the simplicity of youth becomes drivel in the octogenarian; and the *ira brevis* of gout or the delirium of fever require only the establishment of permanent health to place them in the class Vesaniæ. Setting hereditary tendency apart, it is consistent with our present knowledge to extend this measure in proportion as the individual is a member of an educated race, long subjected to training and refinement; as such process modifies the quality and susceptibilities of the mind, if not its power.

But where there is no legal nor social, nor even nosological status implicated, there is a practical value in passing in review the phenomena, or some of them, which may be classed with health or disease, are compatible with either, and sometimes, though of infected origin, subserve useful purposes, or, to continue our illustration, which belong to disputed ground. It is unfortunate that such inquiries should generally be prosecuted under legal auspices. A crime or breach of the law has been committed, or the management of a property is at stake, and it becomes necessary to decide upon the moral condition and rights in the face of punishment or deprivation, and as much in reference to results as to medical principles. The very difficulty which has been encountered in adjudicating where slight or doubtful deviations from the familiar manifestations of mind, where the apparent health and shrewdness of the individual are complicated with eccentricity, or obtuseness, or partial weakness, has led to very broad and rough-hewn distinctions, and to a disposition to disregard all allowance for specialities, and refinement, and delicacy in diagnosis. We encountered a landed proprietor during our noviciate who invariably produced a repeater, put it to your ear, and made it strike. When he had done this, he introduced brief, pithy, pungent pictures of his friends and acquaintance, coarsely and quaintly like, but which were invariably touched off by a dash of the censorial brush which gave them a family resemblance. His neighbour was a liberal man, a great agriculturist, thought right, talked right, acted right, "but," stroking his chin, and twinkling his grey eye, "a d——d scoundrel at bottom." His grandmother was charitable, mild, a supporter of Dorcas societies, village libraries, and the saviour of all the blackguards in the parish; "but," with the same stroke of the chin and the same glance, "a d——d scoundrel at bottom." Was this man a madman or a misanthrope?

In like manner, when the distinction between sanity and insanity is discussed by theologians or moralists, the tendency to examine the mind in the abstract rather than in the concrete, through the individual consciousness, or in comparison with some perfect or lofty example of human greatness rather than with the experience of the whole field of observation, embracing not merely the opposite extremes but the intervening degrees of strength or soundness, is invariably observable. The reluctance to pass the moral eye along the whole of the ascending and descending series of moral and mental phases to and from the Godlike, becomes still greater when it is inquired whether there be any demonstrable boundary line between insanity and crime. It appears, however, incumbent to ascertain, not spasmodically, and when some frightful catastrophe occurs, but as bearing upon the general laws of mind, whether the mental constitution of the dangerous classes be the same, or intrinsically similar, to that of those upon whom they prey. The elements of moral culpability and legal responsibility may be altogether excluded from consideration, and the analysis confined to the relations of the anormal tendencies to physiological science or medical treatment. In the case of murder, it may be fairly held that the greater the atrocity of the deed the greater is the probability that it originated in some detectable departure from right reason. The devout, zealous Boggia, "the darling of the Milanese priesthood," orderly in habits, finical in dress, calm and collected in demeanour, destroyed five victims without any very appreciable motive, without a suggestion of vengeance, and in the same way that a man kills a caterpillar. We do not speak of motiveless crimes; for the destruction of life, the gratification of cruelty, however sudden and transitory the impulse, are motives, but of acts which are foreign to the disposition, inconsistent with the interests and objects of the perpetrator. Yet, if not without motive, it baffles human penetration to discover an intelligible ratiocination or object which could lead a man to "confess that he was party to the murder of one who turns out afterwards to be alive; who, not satisfied with endangering his own life, implicates his own mother and brother in the supposed guilt, so that the whole three perish upon the scaffold—the victims of a fabricated narrative."* The insane, when bloodthirsty, slake the appetite by killing their relations. A commonplace murder, if it stands alone, may be treated accordingly; but if it be committed by individuals who have displayed an incapacity for the rudiments of knowledge, or whose hands have been imbrued in blood, although it may have been the blood of beeves, who went to Waterloo to see the carnage, who have frequented slaughter-houses or executions—like the witty Selwyn or the humane and patriotic Penn—who have spent hours of a springtide day in cutting off the heads of myriads of green lizards, as we have seen a boy do; or sawn through the scaffolding of a house in order to enjoy the fall and crushing injuries of the Irish hod-men, or bribed the poultry-woman to act as her substitute in strangling the geese or bleeding the turkeys, as we have known done by a mature and educated man; the character of the act is materially modified. Calcraft has a shadow. He gloats over every step of the process, from the adjustment of the rope to the last faint quiver of the defunct offender. The motive of such a rational monster may be that of a necrophile, or of a bloodshedder who dares not kill, or who shuns the penalty of killing. Dumollard may have been a savage artist, a vulgar vivisector, as well as a criminal. These circumstances must be kept in view, because the propensity to destroy life may be associated with peculiarity of mental constitution, and because unequivocal insanity is known to follow such manifestations, and is the direct consequence of

---

* *Romance of Forum*, vol. i. p. 14.

vice, dissoluteness, and irregularity. Nor must it be forgotten that certain diseases, such as epilepsy, appear to predispose to derangement as well as to crime, and that lunatics and criminals are often members of the same family. The disclosure of uncommitted crimes would prove an interesting chapter in ethics, and might cast light upon some of the darkest depths of human motive. There might be revealed sanguinary passions and plans defeated by fear or circumstances, unsatisfied vengeance, slumbering but dreaming beside high resolves or a blameless deportment, and imparting perhaps energy to the whole nature. A certain organization, or hereditary proclivity, may thus include potentially, and be capable of being developed into particular diseases—gout, rheumatism, phthisis, rachitis, and contemporaneously into heroism, extravagance, debasement, and nervous derangement. Rickety men are large-headed, crook-backed, often precocious, wayward, never mature on all sides, and more liable to the faults and fallibilities of mankind than the well-developed and robust. Yet Alfred was a "ricketty weakling;" Godwin was a hydrocephalic dwarfling; and lame men have attained to the mountain heights of science and literature. It has been epigrammatically said that had not Walter Scott and Lord Byron been deformed, the one would have been a moss-trooper the other a corsair. But is an imperfect organization ever subservient to perfect mentalization? The mind is made up of external impressions; and it is greater and grander and more comprehensive in proportion to the number and varied source, as well as to the force of these, and to their combination. An eyeless mind is shorn of its natural proportions, and it cannot form judgments so accurately in connexion with vision; it cannot be so responsible when reasoning and feeling as to objects of which it can have no adequate conception. An armless or legless man is morally, as well as physically, mutilated. This speculation is practically admitted with respect to paralysis; but it is doubtful that a jury, whether in or outside a court, would hold a man less capable of recognising his duty or of self-control after than before amputation.

Push the doctrine of latent consciousness, again, to its legitimate conclusion, and there may be in each mind unknown, undiscoverable, hideous errors and incongruities from which it would recoil, but ineradicable by itself; but of which glimpses may be obtained in delirium, in moments of danger, or in dream. Intelligence and geniality of heart, as well as fury and lust, are sometimes roused by wine; and intoxication and the literature of the insane show not so much that eloquence, and taste, and elegance, and excellence in composition, are compatible with delusions and aberration rendering seclusion necessary, but that certain powers appear to be developed, intensified, during and by morbid excitement.

May not martyrs and heroes, the saintly Cranmer, the heathen Mohawk at the stake, have owed their triumphs, not to the nature but to the intensity of the emotion, to the anæsthesia accompanying monomania and monoideaism? They did not suffer because they did not feel. In many temperaments a certain amount of stimulant is required to bring up the intellect to the thinking and acting point. And the balance appears to be delicately hung between courage and pusillanimity, dulness and brilliancy. Cowper's failure might have been averted by a bottle of stout; and the oratorical power of a deceased Lord Advocate of Scotland might have been saved by speaking while the opium stimulus was at its maximum.

Eccentricity, again, premonishes insanity, and is a consequence of insanity. It is one of the vestiges, the effects of the storm after its violence has subsided. It is, moreover, a concomitant of great minds. It has been the immemorial excuse for the vagaries of genius; and there has been found a solution for its absurdities in the abstraction, the inattention of heavenborn minds to terrestrial and trivial things. But it exists in the dull and the stolid, and is the chief mark by which they rise above, or fall below their fellows. A jog-trot man dresses, and furnishes his house, and harnesses his horses in linen; and though he argues the preference adroitly, is smiled at. It was at one time fashionable for youths to be pensive, and desponding, and despairing; to cultivate and exaggerate the sentiment of melancholy, and to affect the sorrows of Werther, solitude, and suicide, as they now do bitter beer and the smoking-room. Renowning in German student life and Bloomerism in America were practical examples of the coveting distinction and gratification by outraging the ordinary rules of decorum and deportment. They were epidemics of eccentricity. Such manifestations of minds accredited sound and trustworthy are often more outrageous and pathognomic of alienation than when that disease is present. A gentleman possessed of a large patrimonial estate, and accounted sane, and exercising the powers and privileges of a Lord of the Manor, habitually crammed apartments in his dwelling-house with hundreds of heads of game, and left them there to rot.

There are hundreds of imbeciles who are born to toil like a squirrel in a cage without pause, or precise object, or reward; there are others who maintain themselves; and both classes pass creditably and innocently, if not unnoticed, through that state of life in which it has pleased Providence to place them. But these men, if transferred to another position, to a wider sphere, like the squirrel in a mill-wheel, would become a laughing stock, or a nuisance, by their wild and extravagant attempts to act as other men, or as they did formerly, or as the removal of poverty or of restraint suggested. Give them a thousand a year or a passion which their limited reason could neither regulate nor usufruct, and they would be styled mad or wicked. There are others who, under the protection of an honoured name, the shadow which conventionalities, retirement, or routine duties afford, or amid the drops of the golden shower, are never found out. Let them be tested, however, by loss of station or wealth, exposed without baldrick, or cloth of gold, and they will shrink in native and naked feebleness and incapacity before the pitiless reverse, from inability to adapt themselves to their new position. There are many men, how many it would startle statisticians and those who trust to the rapid perfectibility of the race to say, jostling us in the ordinary walks of life, discharging duties, acquiring dignities, who are not imbecile, but who labour under various kinds of delusions and mental perversions, who know that they are mad, and whose most prominent and precious health-power is to conceal from others their infirmity. Like Augustine struggling against incontinence, they may acquire strength in subduing or shrouding weakness. Connected with this class are those who are insane without knowing it, and in whom it is not detected, and who attain fame and fortune in virtue of those powers to which disease has given stimulus and force, or of the loss of that balance which others prize so much and undergo a life-long discipline to preserve. Mahomet, and the whole succession of heroic impostors, are examples of this ; and if we believe certain biographers, numbers of pietists have unconsciously surrounded gross hallucinations not only with the odour, but with the nimbus of sanctity.

May theft, again, be a plebeian offence but an aristocratic folly ? A priori, it would appear more natural, if not more pardonable, to steal under the instigation of hunger or want, or the desire to possess, than amid luxury and under the influence of indifference. The wealth of the thief is often the only index of the morality or immorality of the act. Thieves, like Thugs, have a pedigree. They may not, like the Knights of St. John, boast of eighteen quarterings, but they can at least be proved to have been hanged, drawn, and quartered through three generations. We recollect of a Border Club flourishing in the debateable land of the West Marches, into which no member was admissible who could not prove that his grandfather had been hanged for sheep stealing. A semi-royal House, the motto of which is not *vivitur ex rapto*, derived many of its manors and honours in the same moss-trooper fashion by centuries of "rugging and riving." This took place when robbery occupied a different place in the sliding scale of morality from what it does now. Purloining in the present day is, in certain classes, more generally recognised as a premonition, and as a symptom of the ordinary forms of alienation. In such a case the unequivocal and general disorder removes all doubt as to the special act. When it stands alone, the true nature of the proceeding may be determined by the articles taken and the manner in which the abstraction is effected. They may be useless, as when a physician confines his depredations to table-cloths; absurd, as when a lady carries off a chattering parrot from Soho-square bazaar; disgusting, as where a man of fortune cribs and conceals decayed meat. The thief may be a millionaire in one particular object. In one case there were collected by an industrious clergyman, in the active and popular discharge of his duties, thousands of copies of the Bible. We recollect an old woman who confined her robberies to tops, and another who was a *virtuoso* in keys—the key of the College of Physicians, of the county prison—nothing but keys. Lord Portsmouth took snuff-boxes, forks, &c. Silver plate—the object coveted, is generally bright—lace, gloves, suggest suspicion; but if they be returned, if they be stolen when many hundreds of pounds are paid to account, the suspicion suggests rather a feat of dexterity than simple shoplifting. The grotesqueness of the object may avert condemnation. Miss E. Osborn, a fashionable lady, carried off jars of potted meat, and was exonerated; and a stationer appropriated a dirty handkerchief, and was pitied: had it been in the one case embroidered slippers, the choice of Lord W. Poullet, or silver spoons in the other, the result might have been different. The motives assigned for committing such larcenies, from whatever source they may really arise, are manifold, and are calculated to add perplexity to the analysis of the mental condition with which they are associated. A clergyman steals Bibles in order to circulate the Gospel; another takes money from the Offertory in order to secure the better administration of the money for the poor; an antiquary removes a piece of money, unvalued by the owner, in order to enrich a collection of coins; and a poor malefactor was hanged for robbing his master in order to give the money to his mother, having knelt down when about to seize his prize in order to pray for God's blessing upon the undertaking. But theft, even when morbid, may spring from covetousness; from a desire to deceive, to hoard, as a gratification of cunning, as a means to attain an end. Articles are often stolen in order to minister to the insatiable desire for stimulants, and there are then combined either two kinds of depravity, or two of the unacknowledged forms of morbid propensity. It may be argued that if there really exists an uncontrollable propensity to inebriety, as there is admitted to be to homicide and arson, it may be conjoined with any possible moral condition; and that the law should exonerate the individual so actuated from the penal consequences of his conduct, but is justified in depriving him of liberty and in consigning him to an asylum. But until the humane example of the legislatures of other countries be followed in this respect, it is much to be regretted that some separate retreat does not exist where a voluntary, or even compulsory, seclusion could be resorted to: where the drunkard, whether insane in the ordinary sense or not, would be treated as an invalid, subjected to a natural and invigorating regimen and discipline, and inoculated with habits incompatible with intemperance and excess. The absence of such a moral lazaretto, and the obvious injury to society, and the cruelty to the infatuated sufferer arising from permitting free scope to his extravagance, and from then punishing it as a crime, has led a benevolent public officer to suggest that cells should be erected in connexion with public prisons, where the fury of the paroxysm might exhaust itself, but where, of course, the durance would be penitential, not curative. Three forms of anormal appetite for stimulants are encountered. There is, first, the frequent variety in which the long, the excessive, but voluntary and deliberate indulgence of the propensity, gratified it may be in the social circle, and to obtain momentary excitement, to display wit, or imagination, or song, has produced directly mania or fatuity. There is, secondly, the brief delirium immediately succeeding a debauch or a course of dissipation. In both of these forms it will be observed that the act, or habit of intoxication is obviously the cause of the disease; but in the third, the intoxication, or rather the craving for stimulants, for wine, or opium, or more ardent potations, is the symptom, the distinguishing characteristic of the alienation—in fact, the tendency to ebriosity, with impairment of the power of the will, constitutes the disease itself. In the first two species the appetite is created, cultivated under the sanction and by the very act of the will, while the drunkard possesses, or appears to possess, sound bodily health and such intellectual perspicacity and vigour as to be accredited sane, and to be intrusted with the business, the burdens, the honours of life. In the third, the propensity may be morbid—it is certainly instinctive, involuntary. It sometimes originates in infancy or extreme youth, or age, where no preliminary course of indulgence could have merely converted a habit into a disease, where the individuals were recognised and respected as virtuous, rational, and it has happened, abstemious and even ascetic. The paroxysm is developed suddenly; it hurries its victim, in opposition to his best interests and present wishes, into scenes of degradation which he detests, and from purposes in which he delights; it returns periodically, and leaves the mind temporarily weakened and wayward. It may arise without provocation or premeditation while the mind is engaged in intellectual labour, in abstract reasoning, or while under the dominion of the purest and most elevated sentiments; and, in a moment, prostrates and paralyses the most firm resolves, the most virtuous motives, the most colossal obstacles of reputation and interest, and plunges its slave into an abyss of drunken delirium. It may coexist with reasoning power and many acquirements; but there is, especially at advanced periods of addiction to stimulants, a concomitant, and one which may be erected into a crux of the unhealthy origin of the tendency, and which increases the complexity of the analysis by apparently indicating the broad and deep corruption of the disposition—this is untruthfulness, or, if not absolute mendacity, a loss of that reverence for and delicate perception of truth and rectitude which dwell in the healthy upright mind. In this secret, all-pervading influence of a profound lesion is involved the whole dispute concerning Monomania. For it is advanced that a solitary mental state or feeling can for years be distempered and distorted, and yet it may be the product of combinations in other respects sound and sane, and be inextricably connected and mixed up with other intellectual or emotional operations; and yet neither contaminate, nor enfeeble, nor in any degree influence the general mental constitution, nor any other part of it. Philosophy seems, however, to point to the supposition that the effect is rather inappreciable than non-existent.

Gasconading is a euphonious name for that exaggeration, white-lying, or dark defamation, which the imaginative resort to; and versatility is used to defend vacillation of purpose, rapid change in resolution. The giants, however, as well as the dwarfs of our race may be pleased with a trifle, tickled with a straw, and actions of vast import have been determined by inadequate moral causes. Wesley was guided by sortilege, Johnson by an appetite, Fox by a passion; and the most cunning and calculating portions of various communities by South Sea bubbles. There is another colouring of the whole nature by dispositions, which renders it difficult to say whether we have to deal with excess of modesty or want of sense. A noble peer, lately deceased, would have stormed a battery rather than have entered a room, and shrank into a corner in order to avoid the common courtesies of life; yet every town has its tailor whose gratified ambition it is to bestride the weathercock of the tallest spire. We may contrast the politician whose imperturbable gravity was never disturbed, and who enjoyed the *soubriquet* of Single-smile W., with the calm, sagacious, deep-feeling painter of Scottish life, whose laughter seems to laugh at will and wisdom. The timidity of the morally brave Cowper, which prevented him from reading a mere form; the cowardice of the

*beau sabreur* Murat, which made him tremble when the battle began; the objectless timoria of the Sardinian when the wind blows from the marsh; are all as little reconcileable with the laws of mind, or of the particular mind under examination, as the self-possession and equipoise of Blondin.

The sense of order and fitness is so exquisitely strung that one man arranges his beard upon the scaffold as innocent of the treason for which he suffered; another carries snuffers—he was, to be sure, an actor, and might have the footlights ever before him; a third keeps his house topsy-turvy as a source of satisfaction. And there is here not merely the perception of order and beauty, but, as when Mary of Scotland spanned her neck under similar circumstances, or as when the mourner laughs merrily when placing his friend in the earth, that duplicity of thought and feeling, that jesting in death, that mixture of incongruities, which sets the causation of some of our most prominent acts at defiance. Were we to describe a man who insisted upon the trivial routine of life, who wore the same cut of coat for thirty years, who hung up his hat upon the same peg, and always placed his walking-stick upright in the same boot, the portrait would be accepted as that of an eccentric martinet. Were we to use deeper colours, and employ greater breadth, and delineate one who did not love, who did not hate, who did not hope, who did not fear, who did not worship as others do, who separated himself from his fellow-men, and apparently from his God; who was neither earnest, nor enthusiastic, nor heroic, nor grovelling; and who, passionless himself, viewed human nature as he did all other matters qualitatively, the likeness of an undescribed monster or of a madman would be recognised. Yet these two phases were presented by the same individual. He who seemed ruled by the sense of order rather than the laws of our moral nature, whose deficiencies offered a fair field for moral treatment, was the philosopher Cavendish.* The veriest ingrate has loved something—a poet dotes on rabbits, a minister of the crown cherishes leeches; ugliness at one time recommended pug-dogs and China monsters as pets; and yet sudden loathings and antipathies spring up unconsciously, but become permanent and influence the whole conduct in strong minds. But if it be difficult to classify these with the known phenomena of either health or disease, it is much more so if we trace the rise and progress of mind. It grows in general gradually and by obvious acquisition, but upon other occasions it starts suddenly into being and potency, and we are called upon to treat precocity as a disease. And yet could the youth of Walter Scott and of Blaise Pascal be morbid? It is, moreover, remarkable that the early and rapid development

* Wilson's *Life of Cavendish.*

of passions, or purely intellectual faculties, where it is encouraged as miraculous rather than checked as morbid, is apt to lead to nervous maladies or exhaustion. Slow maturation condemns the individual to the fate of an idiot; yet *les enfans arrières* ultimately swell the ranks of sound thinkers. What is true of the general constitution of mind is true of particular powers. Prodigies in music, languages, calculation, point to defect of other faculties by privation. A great mathematician could not repeat the multiplication table; and polyglot Bentley could not write decent English. We see in certain minds torpidity and slowness in elaboration, while the products are sound, brilliant, natural; and in others there is an incessant and feverish activity, which is vain, frivolous, unproductive. If we examine the impressions on the imagination as influencing volition, whether in the volatile disposition alluded to, or in more solid and substantial natures, we meet the castle-building of Hartley Coleridge, the anticipated realization of vain hopes by Law, the visions of Swedenborg, the reverie of Sir R. Peel, the omen of Bunyan, and that extraordinary power of vaticination, exemplified in anticipating events, even in anticipating insanity, all connected with unhealthy conditions of brain, all beyond the dominion of determination and the regulation of the individual, yet not in themselves absurd or irrational. How intimately allied to that which they, in one aspect, simulated, is shown by the fate of M. and C. Lamb, who carried with them a strait-jacket as a provision against that mania foreshadowed in their consciousness; and of Buckland, who executed a testament, leaving the management of his body to certain medical and other attendants after the mind had passed into fatuity, and whose preparations were justified by the result. The glorious cloudland in which H. Coleridge, Law, and perhaps Sir R. Peel indulged, is sometimes the harbinger of a long euthanasia. The visions of the general paralytic of wealth and power and greatness are but day-dreams believed in. At first it was believed that the frightful privilege of dying in ecstatic delusions was confined to the stronger sex. Esquirol conceived that he had met with only one case of a female to 123 males; Foville noticed 9 in 190; Bayle 24 in 182, and Calmiel 1 in 15. This gallantry of statisticians is gradually disappearing, and extends only to the rarity of general paralysis in women. There are, however, many sceptics who declare that they never observed a genuine case of general paralysis in a female. The discrepancy may be regarded as one of the open questions in medical psychology. It may, however, be partly explained by the slovenly mode of diagnosis now applied; for no longer is the symptom originally propounded as pathognomic, the monomania of ambition and aggrandizement, considered important; and in a recent learned work any modification of paralysis, although occurring in melancholia, presenting a particular state of the pupil, is accepted as general paralysis.

There are moments of inspiration, which, short of prophecy, mark a crisis. Let it be the work of love or rage which kindles the excitement, the reason and will become suspended during its continuance, all consciousness of time is abolished, and no recollection of events is preserved. Lord Ferrers may have been hanged according to law, but he was more an unconscious murderer than he who acts so during sleep. Yet he may have indulged his rage, the vision of punishing an offender, and the particular offender may have crossed his imagination in moments of calmness. There is the testimony of orators that their apprehension of what they uttered at certain times was so faint that the *Times* disclosed to them their own eloquence. Mrs. Siddons, and some members of her family, so identified or impersonated characters, that hours passed before the assumed feelings and deportment passed away, and they became themselves again. Methodists have so intensified devotion as to induce convulsion, quietists so excluded external impressions as to remain hours rapt in concentration; and Shakers express adoration by voluntary gesticulations. If the highest attributes of our spirit thus fine away into the lowest, we may be prepared for seeing in the supersensuous conditions of mind anomalies which may be interpreted either physiologically or pathologically. Not only does natural somnambulism develope courage, or a carelessness of danger which enables the individual to scale cliffs, and walk on the summit of houses, and a vigilance of the senses while consciousness sleeps, but it is an index of a highly nervous temperament, and a forerunner of insanity. In mesmeric trances results are arrived at altogether transcending the natural powers of the actors, and yet the least robust, the most facile and ill-regulated and impressionable systems are those most frequently placed in this condition, it matters not whether by objective processes or by subjective impressions. Were the audible oracles, the odours, the visible hand, the ambient wreaths and bodies, and the inferences, all discarded as unreal and the result of imagination, the faith in these, in the conviction of some sensation corresponding to these, are irreconcilable with the known laws either of health or disease. It is impossible to forget that Johnson and Luther saw spirits, that Cazot prophesied, or that Rabbi Hirsch Danemark could read by touching closed books. Now the only distinction, if it be such, between such conditions and popular vulgar madness, is that volition is present in every manifestation. It would entice into subtle disquisitions were the extent to which the will itself may be influenced, or impaired, or perverted by these states of consciousness discussed, but it bears intimately upon the subject that those notoriously insane likewise exercise will over a great number of faculties, in the direction of intellectual powers, in simulating emotions which they do not feel, and in concealing those which agitate them; and that exaggerated will, obduracy, and determination in a degree beyond ordinary capacity, is a characteristic of mental disease. We have known females remain voluntarily silent, and with closed eyes, for eighteen months, in order to prove that they were dumb and blind. There can, however, be no doubt that the power to act advisedly, consistently, and in accordance with personal interest, social laws, and our ultimate destiny is a grand characteristic of soundness. Supposing the removal of a limb, there occur phenomena which cannot legitimately be classed with healthy sensibility. Pain is felt in the limb which has been left upon the field of battle; and although an explanation has been invented by physiologists, the sufferer cannot undeceive himself as to the reality of the pang or of the locality. Sensations, again, equivalent to the aura epileptica, precede or accompany great mental efforts and revolutions; yet luminous circles, coloured objects, articulate sounds, formication, may be lifelong, or they may disappear as if they were the prodromes of disease checked during incubation, but during their continuance interpenetrating and modifying the whole mental constitution; for if intellect be affected by the withdrawal of impressions, it should be disturbed and confused by the reception of those which not only exceed, but transcend, the ordinary standard, and of the existence of which there is no external evidence. There are reasons for believing that besides the gratification afforded to certain dispositions by deception, anomalous sensations may be present in many forms of malingering, and may be associated with hypochondriasis. The tendency is found chiefly in the hysterical: it becomes epidemic, as was seen in Boerhaaves hospital ward; and in the ophthalmia in the 58th Regiment. It occurs in particular families. We have known two sisters labouring under imaginary disease of the spine at the same time, both of whom were cured by the proposal to employ the actual cautery; and it enables the system to defy pain or to become insensible. The skin of melancholics may be made a pincushion, irritated by galvanism, and they give no token of suffering; but we know of a simple, ruddy country girl, who produced sores in her arm which led, in succession, to amputation of a finger, the hand, the forearm, the arm, and for no other intelligible purpose than to obtain the shelter and ease of a hospital, or the sympathy which her protracted illness elicited. Soldiers have a traditional pharmacopœia for purposes of simulation; they have receipts for producing hernia; they induce ophthalmia by the aid of corrosive sublimate or bluestone, temporary amaurosis by belladonna, vomiting by swallowing air or tobacco: they imitate the more popular aspects of insanity, and they are flogged, perhaps justly, as impostors; but the idea which suggests, the resolution which sustains, and the agony which that entails, all bespeak a state of the nervous system which often passes into self-delusion and crime, and however unreal in itself is often accompanied by affections such as cephalalgia, neuralgia, chorea, and rheumatism, which are admitted to be of physico-psychical origin. Hysterics and sentimentalists, even when they do not fabricate maladies, make a capital of their indisposition, whatever it may be, to cloak the real source of crime, or selfishness, or sensuality, or to escape the censure which spontaneous evil, and without such mitigation, might incur, and settle a difficult question by referring them to a common origin.

It is supposed that St. John Long believed in his discovery, that the scars upon his breast bore evidence of the application of his nostrum; in fact, his success in winning the confidence of others, and those of educated if not strong mind, presuppose sincerity, for there is earnestness in credulity as well as in truth. The next step, it is obvious, is where a delusion or a morbid impulse coexists with a vigorous understanding, is known as an error, but cannot be expelled nor separated from the ordinary course of association or feeling. Either vision or audition convey impressions where none exist, as when Nicolai saw troops of his friends in the empty room, or Andral the corpse; or correct impressions lead to irrelevant or inconsistent subjective mental states, as when Bernadotte actually saw the old woman in the red cloak, but interpreted her appearance as a warning: or as a patient of our own, whenever she approached plate-glass was impelled to break it, when in church she was tempted to shriek aloud, and when intrusted with a child felt a strong propensity to cast it down; yet possessed health sufficient to divulge none of these thoughts and to resist all. There may be sought in the limitation and localization of disease a vague elucidation of these phenomena; but we are driven from this by the observation that one organ of sense may remain faithful while the other betrays. A man has heard with one ear a voice which is inaudible to the other; and which the silence perceived by the other, and corroborated by other senses, goes far to show cannot be heard. Different articulate sounds have been heard by different ears at the same time; and a real and a fancied sound by both ears at the same time. The perception of half objects has occurred in fevers and towards the close of life, as in Wollaston, who, however, when a bystander remarked that his mind was gone, made a signal for paper and a pencil, wrote down some figures and cast them up, almost in the act of expiring; and we were consulted by a lady two years before her death, who, in twilight and darkness, saw every lamp, fire, and luminous object as if there had been three, placed at the angles of a triangle. This person was of average intelligence, and truthful disposition. She might, at the time, be labouring under ovarian disease, but died comatose.

These observations may serve to show how vast is the unappropriated territory in the realm of thought: how indistinct is the horizon as seen from either side; how various are the rights and privileges which may be fairly claimed by contending parties, and how irregular and difficult is the arena upon which the contest must take place. Yet there is reason to believe that though the line of demarcation may altogether disappear, and though it might be agreed that not even a nominal boundary should exist, the interests of mankind would not be compromised, nor the progress of human knowledge impeded.

---

